# Surgery

**Published:** 1858-11

**Authors:** 


### SURGERY.

### CASES ILLUSTRATING THE TREATMENT OF SPINA BIFIDA.

CASE I.—LARGE LUMBAR SPINA BIFIDA; TREATMENT BY LIGATURE; DEATH. Under the care of *Mr. Paget.*—Alice Bagster, the subject of the following case, first came under our notice on June 24, 1854, at the City Hospital for Chest Diseases, where her mother was in attendance as an out-patient on account of phthisis. She was then aged three months, and a remarkably stout, healthy-looking infant. Over the lumbar spine was a tumor the size of her head. There could be no doubt about the diagnosis; there was free fluctuation; the tumor was transparent, and its communication with the spinal canal might be proved by making pressure upon it, when the fontanelle, ordinarily depressed, filled out and became prominent. The child was good-tempered, appeared intelligent, and had a well-formed head. It had perfect use of both lower extremities, but there was a slight, and perhaps doubtful, degree of inversion (varus) of the right foot. No symptoms of nervous disease had ever been present. The mother stated, that when born the tumor was about the size of a large fist, and her impression was that it had increased much more than in proportion with the infant's growth. It was now nearly as large as an infant's head; the circumferential measurement was thirteen inches, that over its highest part seven inches and a half.

A consultation on the case took place, at which Dr. Bennett, Mr. Hilton, and the writer, were present. Dr. Bennett was not inclined to attempt any radical measure, and suggested the trial of iodine counter-irritation externally, together with the use of mechanical support to the sac. Mr. Hilton related a case which had occurred to himself some years ago, in Guy's Hospital, in which he had dissected away the greater part of the cyst, and attempted to unite its edges by means of sutures. The result, inasmuch as death from spinal arachnitis had ensued, was not encouraging to the trial of similar means. The propriety of practicing occasional puncture of the sac was also discussed, and finally it was decided that a gutta-percha case should be made to be worn well-padded over the tumor, and if, in the course of time, an irrepressible tendency to increase should be shown, that puncture should then be resorted to. It was accordingly explained to the mother of the patient that the best to be hoped for was, that the child might grow up with the tumor no larger than its present size. This intelligence did not seem agreeable, and a strong desire was expressed that at any risk a cure should be attempted.

On July 16th, we met the little patient and her mother in Queen's-ward, St. Bartholomew's Hospital, where she had been admitted on the previous day under the care of Mr. Paget. The parents were now decided on having some operation attempted, and stated that they would prefer even a fatal result to the altenative of life with so serious a deformity. The size of the tumor had not materially increased, and the child's condition was much as above described. The tumor measured thirteen inches round its circumference, and eight over its greatest height. Pressure did not diminish its size or produce any appreciable disturbance of the nervous functions. Mr. Paget stated that the absence of any effect from pressure, and the possession of full nervous power over the lower extremities, induced him to believe that the neck of the sac must be small, and that the sac itself did not probably contain nerves. A consultation on the case was held, and Mr. Lawrence and Mr. Stanley having expressed opinions in concurrence with that of Mr. Paget, it was decided to attempt a radical cure: the great risk incident to such a procedure was fully explained to the parents. The plan which Mr. Paget determined to practice was the subcutaneous division of the neck of the sac by means of a ligature. It was hoped that by this means the communication between the sac and the spinal canal might be obliterated by the effusion of lymph which would be caused.

On July 26th, the operation was accordingly performed. The child having been put under the influence of chloroform, a strong double silk ligature was passed subcutaneously around the tumor, the ends being finally brought out in the middle of its upper part. The ends having been loosely tied were attached to a band of elastic webbing, and the latter fixed in a state of tension, by means of a bandage passed round the chest. It was hoped that the elastic band would gradually drag the ligature through, and so make a section of the base or neck of the tumor. The performance of the operation was not attended by any difficulty. During the three days following it the child suffered no symptoms other than restlessness, apparently from the pain which the ligature gave.

On the second day there had been a little redness around the border of the tumor; but this had almost disappeared on the third. The ligature had been drawn out at the rate of half an inch daily; there was free suppuration along its track.

On the fourth day the infant appeared feverish, restless, and ill, and rather less of the ligature was, in consequence, drawn out.

On the fifth day the child's condition was much the same. Some clear fluid, as if from the sac or spinal canal, was flowing from the wound at which the ligatures escaped. It appeared as if two or three drachms had so escaped; but the sac was still tense, and its contents quite translucent; it was not reddened or hot.

During the sixth and seventh days the child continued in much the same state, but got more feeble. At times it appeared much sunken, and almost insensible, and the mother believed that it could not see. No convulsions occurred. Throughout it had taken the breast greedily, and still continued to do so.

On the eighth day death occurred. For some hours before, the child's state had been one of almost insensibility.

Permission to make a post-mortem examination having been at first refused by the parents, and the body removed from the hospital, three days elapsed before the inspection was obtained. It was ultimately made by the writer, at Mr. Paget's request. Only the tumor was allowed to be examined.

The tumor was much less tense than during life, and its lower half, and, indeed, the parts about its base generally, had acquired a remarkable feeling of solidity. Compression of the tumor did not not decrease its bulk, but there flowed from the wounds a small quantity of thin, serous pus. A longitudinal section of the whole mass having been made nearly down to the vertebræ, a relation of parts, as represented in the appended diagram, [which we have been compelled to omit.—EDS.] was found. The larger cyst was about capable of holding a duck's egg. It was lined by a smooth, glistening membrane, of fibrous structure, and which could be easily peeled away from the structures with which it was in contact. This membrane, when held up to the light, was only very partially transparent; but there were no evidences of its containing nerve-trunks in any part. The cyst was quite whole in every part, and contained a perfectly clear serous fluid. At first sight it did not appear to communicate with the spine; but, on probing, an opening, covered by some valve-like folds of its lining membrane, was discovered, which led into a smaller cyst. The latter cyst about equaled a half-walnut in size; its base opened freely into the spinal canal, and the trunks of nerves passed out therefrom in a curved direction, and adhered to its walls. There were no traces of the results of inflammation in either cyst or in the spinal canal, excepting that in the latter the small vessels were deeply injected. The laminæ and spinous processes of the last two lumbar vertebræ were wanting. Surrounding the cysts in every part, but especially abundant behind, was a thick layer of dense granular fat, and in this the track of the ligature had run. The ligature had nowhere quite touched the walls of the cyst, but on the right side it was very near to them; posteriorly it was fully half an inch distant. Imbedded in the fat over the sides of the cyst were two small plates of cartilage, one on each side, evidently the displaced and undeveloped laminæ of one of the vertebræ.—*Medical Times and Gazette*, July 24, 1858.

CASE II.—LUMBAR SPINA BIFIDA OF CONSIDERABLE SIZE; SPONTANEOUS CONTRACTION; DEATH FROM HYDROCEPHALUS. Under the care of *Mr. Lawrence.*—The coexistence of hydrocephalus with spina bifida is far from infrequent, especially in the worst class of cases. The frequency strongly supports the opinion that these defects of development are due to intra-uterine arachnitis, and are not mere arrests of vertebral ossification. Thus the effusion into the sac of the spinal cord takes place in consequence of a transitory inflammation at a period of fœtal life prior to the firm junction of the laminæ with their corresponding bodies. By this effusion the laminæ are displaced so wide that union is rendered impossible, and a permament defect is caused. In support of this theory of the formation of a spina bifida, we have the circumstance, that plates of cartilage, constituting the laminæ, are not unfrequently found in the soft structures overlying the cyst. This was observed in the case just recorded. Another fact in support of the same view is, that now and then, when hydrocephalus and spina bifida are coexistent, the one will subside greatly while the other increases. This was observed in the following case:—

Anna B., a puny child, aged two months, attended for some weeks, during 1853, as an out-patient under Mr. Lawrence's care, on account of a spina bifida over the lower lumbar region. The tumor was the size of a child's fist, and the skin in the middle was thin, and at one time threatened to give way. There was imperfect talipes varus of both feet. From birth the head had been noticed to be large, and at the age of one month it began to increase rapidly. Mr. Lawrence declined to adopt any operative measures, and the tumor was merely protected by a pad of cotton-wool. The hydrocephalus increased to such an extent that the child was, after a time, unable to be brought out, and her case was, with Mr. Lawrence's permission, afterwards watched by the writer and his friend, Dr. D. H. Tuke, at her own home. No active remedial treatment was adopted, and the head continued slowly to enlarge. The spinal tumor, however, shrank away, the integument over it becoming thick and wrinkled. When at length the infant died, (at the age of nine months,) its position was only marked by a piece of thick, very brown skin, overgrown with short, coarse hair. On laying it open no fluid was found in what remained of the sac, the cavity of which was very small indeed. The lateral ventricles of the brain were found to be laid into one by the destruction of the septum lucidum, and contained between one and two pints of clear straw-colored serum.

It had been noted in this case that there was no reason to believe that the lumbar sac communicated directly with the fluid in the head, as pressure upon the one did not in any way influence the other. At the autopsy, the canal in the centre of the cord, which, in certain cases of this class, is found open, affording a free channel of communication between the arachnoid cavity of the cord and the cerebral ventricles, was found obliterated. To its obliteration, no doubt, was due the fact that cyst contracted while the ventricles were filling. An interesting example of the reverse was under the notice of the writer about a year ago, and was recorded by him in the last volume of the *Pathological Society's Transactions*. In it the spinal tumor increased until it gave way, while the cerebral distension was also steadily advancing. The autopsy demonstrated a most free communication between the two.—*Ibid.*, July 24, 1858.

Cases III. and IV.—Spontaneous Recovery from Spina Bifida.—The particulars of two cases have been mentioned to us in conversation by Mr. Wormald and Dr. Moore, in which spontaneous shrinking of the spina bifida cyst took place. Neither gentleman, however, was in a position to give us more than a few facts from memory; the cases are, however, of too much interest to be passed over in silence. In Mr. Wormald's, the patient was a male infant, who attended as an out-patient at St. Bartholomew's. The tumor from the first was a very small one. It was situated over the lumbar region. A metal shield (lead) was constructed to protect it, and by means of cotton-wool within this and a bandage over it, moderate pressure was kept up. The cyst gradually shrunk until it became imperceptible, and the skin of the same level as that surrounding it. The skin remained somewhat thickened. Mr. Wormald saw the child last when it was between two and three years old, at which time it remained quite well.

In Dr. Moore's case Mr. Wormald also was consulted. The tumor was over the lumbar spine, and the size of a large egg. Within a few months of birth it ulcerated and oozed a great deal, after which it very gradually lessened, and finally disappeared. It had been throughout supported by a leather pad and cotton-wool.—*Ibid.*, July 24, 1858.

CASE V.—SPINA BIFIDA IN THE ADULT; PUNCTURE; OPISTHOTONOS; DEATH FROM SPINAL ARACHNITIS. Under the care of *Mr. Tatum*.—Llewelyn M., aged twenty, a Welshman, was admitted into St. George's Hospital in the latter part of 1856, on account of a pedunculated tumor over the sacrum. He could not speak English, and the account obtained was imperfect; but it appeared certain that the tumor had existed from birth, and had gradually increased in size. There had never been any pain or disturbance of the nervous system, excepting on two or three occasions, at distant intervals, when the skin had ulcerated. At these times it was said that involuntary movements of the limbs had been noticed, and the fæces had passed unconsciously. The tumor was larger than a man's head, and was attached by a broad, short peduncle over the middle of the lower part of the sacrum. Fluctuation was distinct. The skin was ulcerated in two or three places from the friction of the clothes, and was also crossed by numerous large tortuous veins. The tumor caused no inconvenience, excepting from its unwieldy bulk, and the man seemed to be in good health. He was, however, very anxious to have something done which might rid him of his incumbrance.

In the first instance an exploratory puncture with a grooved needle was made. The fluid obtained was clear, and of a light-brown color, differing from blood-serum in containing much less albumen. It was now determined, after consultation, to try the effects of tapping.

A few days after the puncture a trocar was introduced, and about half a wash-hand-basinful of fluid withdrawn. The tumor was by this means much reduced, but still remained nearly the size of a head.

On the second day after the tapping erysipelas appeared around the puncture. Pain was also complained of in the head and abdomen, and it was noticed that the head was kept drawn backward. The pulse was rather feeble, but there was no marked constitutional disturbance.

On the next day, however, the head was still more retracted, and the urine and fæces passed involuntarily. No muscles were rigid, excepting those of the back of the neck. The opisthotonos increased, so that, on the third day of the attack, the head was noticed to be so much pulled back as to make almost a right angle with the back. This position was not altered during sleep.

On the sixth day after the tapping the tumor gave way, by the ulceration of the wound made by the trocar, and its contents were evacuated. The erysipelas had by this time passed off, but the opisthotonos had continued, and the man's strength had rapidly failed. Death took place the same evening. The treatment had consisted in the exhibition of the bichloride of mercury.

At the autopsy the lining membrane of the sac was seen to be highly vascular, and coated in places with lymph. At the upper part of the neck of the sac was an aperture, apparently in the situation of the natural orifice of the sacral canal, and leading into the sub-arachnoidean space. The bones seemed perfectly ossified. The membranes of the cord were thickened, and from their lower extremity to the mid-dorsal region contained a collection of pus. The cord itself, where surrounded by pus, was much softened, but in others was quite healthy. None of the spinal nerves passed into the sac. There was a large quantity of cerebro-spinal fluid, which was opake, and of a dull-gray color. The brain and other viscera were healthy.

We have condensed the above account of this interesting case from the report given of it by Mr. Holmes in the *Pathological Transactions*. The case proves —1. That the subjects of spina bifida may attain adult life; 2. That the enjoyment of good health is compatible with the existence of a large tumor of this kind; 3. That spontaneous ulceration and drainage from the cyst may occur without inducing serious consequences; 4. That artificial evacuation may produce a fatal result.—*Ibid.*, July 24, 1858.

CASE VI.—SPINA BIFIDA, WITH HYDROCEPHALUS; PARACENTESIS; DEATH.—We may here suitably mention a case, though not in hospital practice, which may be found in vol. viii. of the *Transactions of the Pathological Society*, which was treated by puncture. The infant was born with a sacral spina bifida of considerable size. Its frontal suture was also open, measuring half an inch at the nose, and fully two inches at its widest part. Both feet were slightly incurved, the right most so, the right leg being atrophied. The child was well nourished, and did not appear to suffer any inconvenience from the tumor, except when pressure was made on it. Puncture was practiced when the child was a week old, and was repeated once or twice afterwards. The child died in the ninth week, having previously had convulsions. It does not appear from the narrative that any ill consequences were attributed to the puncture; on the contrary, it is stated that temporary relief was afforded. The case was under the care of Dr. W. Ogle.—*Ibid.*, July 24, 1858.

CASE VII.—LARGE SACRAL SPINA BIFIDA IN A HEALTHY ADULT. Under the care of *Mr. Hilton.*—Rebecca W., aged twenty-three, was under Mr. Hilton's care, in Guy's Hospital, during April, 1853. The ailment for which she was admitted was a fistula near the anus, which was believed to communicate with carious disease of the end of the coccyx. The circumstance which gave interest to her case was, however, that there existed a large spina bifida. The tumor, which was as large as two fists, had a broad peduncle of attachment to the left side of the spines of the sacrum, and itself occupied the upper part of the left buttock. To the touch it felt tense, and there was free fluctuation. The skin over it was loose, and might easily be pinched up. On taking the tumor in both hands, and compressing it, she complained that a disagreeable sensation of fullness in the head was caused, and that flashes of light were seen before the eyes. Many trials were made, and these symptoms were invariably produced. She was a married woman, and had borne two children, her labors having been, she said, without any unusual occurrence. She was obliged to be very careful how she sat down, for fear of injuriously pressing the tumor, but the sensations in the head always gave her warning when it was squeezed. She had never suffered any further inconvenience from it beyond these peculiar head-sensations, excepting on one occasion, when, having fallen out of a swing, and struck the tumor, she was for a fortnight unable to stand, and during the first part of the time could not even move her arms. Having kept her bed, however, for some weeks, these symptoms gradually passed off. She was of sanguine temperament, cheerful, and of active habits, and had, on more than one occasion, taken long sea-voyages. It did not appear that in infancy the tumor had caused any symptoms. It had been noticed immediately after birth, and several medical men were then consulted about it. At a subsequent period she was taken to the late Mr. Key, who strongly advised her that no interference should be permitted. There had never been any degree of clubfoot. The fistula for which she now came under care, had resulted from an abscess near the rectum, which had formed during her homeward voyage from America about two months before her admission. It did not appear to be in any way connected with the spinal tumor.

The only parallel to this case in respect to the large size of the tumor, the entire absence of symptoms, and the age of the patient, which has ever come under our notice, is the one cited in a previous part of the series, (*Case* 5.) In the present case, the tumor, from its lateral position and smaller size, caused much less inconvenience than was experienced in the other. There was no temptation to interfere; for the woman really suffered nothing from it, and was competent to all the duties of her station, and the full enjoyment of life. Such instances are, doubtless, very rare, though probably not quite so nearly unique as some might suppose.—*Ibid.*, August 21, 1858.

CASE VIII.—TUMOR, SUPPOSED TO BE A SPINA BIFIDA, OVER THE SACRUM OF A HEALTHY ADULT. Under the care of *Mr. Hutchinson*, at the Metropolitan Free Hospital.—In neither Mr. Hilton's nor Mr. Tatum's cases, above recorded, could there be any doubt as to the diagnosis. The tumor was in each of congenital origin, and there was direct evidence that its sac still communicated with the spinal theca. In the following, the tumor was of much smaller size, and the history of its having existed at birth being wanting, there still remained some doubt as to its nature. It presents, however, features of sufficient interest to be worthy of record, notwithstanding these detractions from its value.

John E., aged thirty-two, a fairly muscular man, in good health, applied, on April 14, 1857, for advice respecting a tumor which existed over the spinal processes of the middle of his sacrum. It was exactly central, and about the size of a duck's egg, but more rounded. It had an evident peduncle of attachment to the vertebræ, and gave to the finger a not very distinct impression of fluctuation. It felt, indeed, as if fluid were confined within thick and tense parietes. The solid structures appeared of varying thickness in different parts, and on the right side was what felt like a plate of cartilage. The tumor was exceedingly tender, and darting sensations in the back and down the thighs were complained of when it was pressed. The man said that he had suffered a great deal from it, both on account of pain arising spontaneously, and also from the effects of its being accidentally squeezed. He was very desirous that something should be attempted for its removal, if it could be done safely,

The history given was, the tumor had not been noticed until he was twelve years old, that it had increased slowly for the first year or two, and had latterly remained quite stationary. Two years before it was first discovered he had been under care in the Oxford Infirmary on account of a swelling on one knee, for which the knife was used, or, as he said, "a tumor was taken out." Thirteen years ago he was an in-patient at St. Bartholomew's, under Mr. Vincent's care, on account of the spinal tumor. Many consultations took place as to its nature, and he was finally told that it was in all probability connected with the spine, and, that to meddle with it would kill him. From that time to the present it had not, as far as he could tell, altered either in size or solidity.

Notwithstanding the doubt which hung about the case on account of the absence of congenital history, yet, after careful consideration of its various points, Mr. Hutchinson thought the probability of the tumor having a connection with the spinal canal was too great to allow of its removal being attempted. The man was accordingly advised not to have anything done, but to wear over it a gutta-percha cap padded inside with cotton-wool as a protection against chance pressure.

Supposing the tumor in this case to have been a spina bifida, it is probable that it was overlooked in infancy on account of its being of very small size, and that it did not materially increase until shortly prior to its first discovery. Such a history of the course of these tumors is however very unusual.—*Ibid.*, August 21, 1858.

CASE IX.—PEDUNCULATED SPINA BIFIDA; OPERATION BY LIGATURE; DEATH. Under the care of *Mr. Erichsen.*—In June, 1855, a healthy infant, aged six weeks, was admitted under Mr. Erichsen's care into University College Hospital, on account of a lumbar spina bifida. The tumor was the size of a small orange, fluctuated freely, and was translucent. The pedicle by which it was attached to the lumbar spine was not thicker than a finger. Although the tumor could not be materially diminished in size by compression, yet there seemed little reason to doubt that it communicated with the spinal theca. Mr. Erichsen adopted the ligature plan of treatment, and having passed a strong double silk through its base, tied it in two halves. A large portion of the tumor sloughed away within the week, and, up to the eighth day, the infant seemed doing well. It died, however, from the irritation caused by the operation, within the fortnight. In this case the infant was, at the mother's wish, treated, at some disadvantage, as an out-patient. No post-mortem was, we believe, obtained.

The case is interesting as an example of a very narrow pedicle in spina bifida. In all probability, the channel of communication with the spinal canal was very small indeed. One of nature's processes of cure is to close this channel altogether, thereby leaving the cyst as an isolated shut sac. Many of our readers will recollect the details of an important case brought before the Medico-Chirurgical Society about a year ago by Mr. Solly, in which a pedunculated cyst depending from the neck had been excised. At the time of the operation there was no communication whatever with the spinal canal; but in infancy it had been considered, no doubt quite correctly, to be a spina bifida. This mode of cure, although applied to a morbid condition instead of a natural one, is identical with that by which the tunica vaginalis testis becomes shut off from the peritoneal cavity. The cure, when it takes place, is probably well advanced during intra-uterine existence.—*Ibid.*, August 28, 1858.

CASE X.—LUMBAR SPINA BIFIDA; OPERATION FOR RADICAL CURE; DEATH. Under the care of *Mr. Borlase Childs*, at the Metropolitan Free Hospital.—In November of last year a healthy infant, aged one month, was admitted under Mr. Childs' care, with a lumbar spina bifida of about the size of a Seville orange. It was situated exactly in the median line, and over the spinous processes of the fourth and fifth lumbar vertebræ. It was translucent, and fluctuated freely. By pressure it was easily emptied, and the finger could then be inserted into the orifice of communication with the spinal canal, and while retained there prevented refilling of the sac. The mother stated that the child had always enjoyed good health, and was not unduly restless. It had been born at the full time, and presented no other ailment. There was no degree of talipes. The tumor had not materially increased since birth, but the integuments overlying it were very thin. The head was of natural size, and no cerebral symptoms had occurred. The parents were very anxious that a cure should be attempted, and were quite willing that any risk should be run by which such a result might be hoped for.

The operation devised by Mr. Childs was of a somewhat novel character, being a modification of the procedures of Dubourg, Tavignot, and other French surgeons, who cut away part of the walls of the sac, and then united the skin across. Mr. Childs thought that much danger might be avoided by not opening the sac itself, and proposed to dissect off the skin and remove its thinnest and central part, and then having tucked the serous membrane into the spinal canal, to unite the margins of the skin over it.

The child was put under the full influence of chloroform. The dissection of the skin away from the sac was found, on account of its thinness, and their close union, to be a matter of extreme difficulty; and before its completion the serous membrane had been pricked in one or two places, and a few drachms of clear spinal fluid had escaped. This escape made the return of the serous sac into the spinal canal more easy. An ellipitical portion of the thinnest integument having been cut away, the sound skin was brought together on hare-lip pins, and a bandage and light compress applied. The child had borne the chloroform well, and recovered from its influence very satisfactorily.

November 10 (the day after the operation).—The infant had slept fairly, taken the breast well, and showed no unusual restlessness.

11th.—An occasional twitching of the muscles of the face and arms was noticed, and the child had a peculiar startled countenance. The anterior fontanelle was bulged. The skin perspired freely, and the bowels were open. The edges of the wound appeared to be united. A powder, containing a grain of calomel was prescribed, and it was ordered that the infant should be kept in a dark room, and have an evaporating lotion applied to the scalp.

On Nov. 12, the fourth day after the operation, death took place rather unexpectedly. No vomiting, nor any positive convulsions had occurred between the time of the operation and the date of death.

No post-mortem could be obtained, but there was no doubt that death was due to spinal irritation, and in all probability conditions similar to those noted in the following and preceding cases would have been found.—*Ibid.*, August 28, 1858.

Case XI.—Large Lumbar Spina Bifida; Commencing Hydrocephalus; Paracentesis of the Tumor; Death. Under the care of *Mr. Hutchinson*, at the Metropolitan Free Hospital.—A young infant, otherwise in fair health, was admitted during 1856, under Mr. Hutchinson's care, on account of a spina bifida in the lumbar region, as large as a moderate-sized fist. There was no club-foot, or other deformity, but the head was rather large, and the fontanelles were widely open. The tumor fluctuated freely, and was translucent. By pressure it could be considerably diminished in size, but refilled as soon as the hand was removed. The skin over it was thin, but quite sound. Its base was as large as any other part. It was in the first instance not intended to adopt any surgical measure for its treatment, and a cup of gutta-percha having been fitted to it as a protector against injury, the parents were apprised of the danger incident to interference, and were told that the best that could be hoped for was that it would not increase.

For several months the child attended regularly as an out-patient, and all did well, no perceptible increase of the tumor taking place. When, however, at the age of seven months dentition commenced, both the tumor and the head underwent rather rapid increase of size. The infant also suffered from slight convulsions, diarrhœa, and other signs of cerebral irritation. At length, the tumor having become tense, almost to bursting, it was determined to relieve it and the head also by tapping. This was done; a small exploring trocar was introduced, and about eight ounces of clear spinal serum drained away. The canula having been removed, the puncture was covered by plaster, and the whole tumor, now flaccid and loose, was supported by a pad of cotton-wool placed inside the gutta-percha shield. The infant had borne the operation well, and during that and the following day it seemed relieved by it rather than otherwise. Subsequently, however, symptoms of spinal irritation, restlessness, twitching of the limbs, etc., developed themselves. There was nothing, however, to excite unusual alarm, until on the tenth day convulsions occurred, in one of which ultimately death took place.

At the autopsy, the cyst was observed to have refilled but very little since the operation. It was still flaccid, and almost empty. On laying it open, however, it was found to contain several tablespoonfuls of creamy pus; and adhering to its lining membrane and to the arachnoid of the spinal canal were many films of effused lymph. Only a few nerve-twigs of very small size could be traced passing out from the theca into the interior of the cyst. The inflammation of the spinal membranes did not extend more than about two inches from the entrance to the cyst upward toward the head.—*Ibid.*, August 28, 1858.

Case XII.—Large Spina Bifida; Puncture; Bursting of the Tumor; Death. Under the care of *Dr. Barnes*, at the Royal Maternity Charity.—

May 11th, 1858.—A full-sized male child born yesterday. There is a tumor extending from the upper part of the sacrum to the dorsal vertebræ, about two inches and a half in diameter. The circumference is formed by a projecting ridge or roll of skin. The covering of the tumor does not present the appearance of skin, but of a thin membrane. The tumor is irregular, presenting elevations and depressions; and in color and form resembling a knot of congested piles. The spinous processes of all the lumbar vertebræ seem deficient.

On the following day the sac became distended with serum to the size of an orange.

On the 14th, the distension had increased, and threatened to ulcerate the sac, which was highly inflamed and excoriated in patches. The sac was now punctured, and two ounces of straw-colored fluid escaped. On the 15th, the sac had filled again; it was so tense that on the 16th it burst. After bursting, the child was frequently convulsed. On the 17th, I witnessed a fit. The arms were stretched out forward, and rigid. The child cried as if in pain and terror, and there was a little foam from the mouth. The mother thinks the legs have been powerless from birth.—18th. Died after repeated convulsions, which were limited to head, arms, and trunk. The legs seemed paralysed. No autopsy.—*Ibid.*, Sept. 11, 1858.

2. ACCOUNT OF SOME TRIALS MADE TO FACILITATE THE REMOVAL OF STONES FROM THE URINARY BLADDER; EXTRUSION WITH THE FINGERS; LANDING-NET. By *Professor A. Buchanan, M.D.*, one of the Surgeons to the Glasgow Royal Infirmary.—Among the advantages which attend the rectangular operation of lithotomy, one is to render more easy the extraction of the stone in the ordinary way, with the aid of the forceps. This depends upon the nature of the operation, which, by diminishing, as far as can be done, the distance between the opening made in the bladder and the external aperture of the operation wound, brings the stone more within reach, and thus facilitates the ordinary manipulations for extraction. Of the extent of the facilities so obtained, those only can judge who are familiar with this operation, and have had opportunities of comparing it with the old lateral operation, which it has for many years past superseded in and around Glasgow—the only field on which hitherto the two operations have come into competition with each other. Still, however, with these facilities, there is usually more delay and more difficulty experienced in laying hold of and extracting the stone, than in making the incision necessary to get at it. It is therefore chiefly to this second stage, that any attempt to simplify and improve further the operation of lithotomy should at present be directed.

Influenced by these views I was led, last winter, to make trial of various methods of removing stones from the bladder; and I propose at present to bring under the notice of those who take an interest in such researches, and understand the capabilities of the new mode of operating, one or two of the methods which I tried, and thought most promising of good results:—

*Extrusion with the Fingers.*—The first method is, perhaps, more curious than useful. I found that after making the usual incision, according to the rectangular method, it was quite possible to remove certain stones with the fingers alone, without the aid of any instrument whatsoever. Stones of a spherical shape and smooth surface, like marbles, even though of large size, could readily be so removed; but to stones of a different shape, and of rougher surface, this method was found to be inapplicable, unless the opening in the bladder were of large size. The following remarks will render the mode of manipulating easily understood:—

The forefinger of the right hand, introduced into the bladder through the operation wound, readily reaches the stone, and has it so much under command that it can easily be brought down into the triangular space at the neck of the bladder, and placed there so that its longest diameter—if it be not spherical—may be parallel to a line at right angles to the middle of the incision of the bladder. This is the position most favorable for the extraction of the stone, and by the pressure of the forefinger it can be retained steadily in that position. Holding it therefore firmly, the fore and middle fingers of the left hand are introduced into the rectum, and passed up beyond the prostate, when, upon pressing them forward, the stone is distinctly felt by them; and it is so firmly grasped between these two fingers and the forefinger of the right hand as irresistibly to suggest to the mind the attempt to extrude it from the bladder, by means of the two fingers of the left hand pressing it from behind, while the forefinger of the right hand guides it outward, and regulates the direction of the pressure.

In this way, as stated above, smooth and spherical stones are readily removed, but only these, unless with a larger incision than usual. The process is therefore of such limited utility, that it would not have been worthy of the space the description occupies, had it not been that to bring the stone and fingers into the positions just described is often an important preliminary to other methods of extraction;—as to that next to be described, and to the ordinary method of extraction with the forceps—if it be the latter, instead of grasping through the bladder to find the stone, and attempting to seize it in whatever position it lies, the forceps is at once laid over the stone in a known position, and the stone is thrust between the blades by the fingers of the left hand, and laid hold of in the most advantageous direction; that is, with its long diameter parallel to the blades.

*Landing-Net.*—The great majority of stones cannot be extracted by the fingers alone, but require the aid of an instrument. Of various instruments for the purpose which I had constructed and tried, the one which seemed to me to answer best the ends for which it was devised, I named a *landing-net,* from its resembling very much, in its mode of operation, the tackling of the same name, with which the angler secures the fish he has hooked and brought to the water's edge. I had this instrument first made with a single handle, and an oval elastic rim of whalebone, to which the net was attached. But finding this not to answer well, I returned to a construction differing only from that of the common forceps, furnished with a net, in the modifications to which I subjected it. The handles are in no respect different, but the blades have altogether changed their character, as they are no longer intended to grasp the stone, but merely to open and shut the mouth of the sac which is attached to them. In conformity with this new destination, they are rounded and attenuated, so as to resemble stout stocking-wires. They are curved, so as to form together, when closed, an oval orifice to the sac; and they terminate in two rounded knobs, like peas, which prevent them from doing any injury when they are introduced into the bladder, or when opened and shut within it.

An instrument of this kind lays hold with great facility of stones of the size of those usually met with in the bladder. On placing the mouth of the instrument over the stone, and making downward pressure, the mouth tends to open spontaneously to admit the stone; and next, the hand holding the instrument, which had at first yielded to, and gently assisted the distending force, now closes the mouth, so as to include and secure the stone within the sac. In this way, without any further preliminaries, a stone may be laid hold of within the bladder, the metallic knobs detecting the place of the stone; and these knobs being carried to the farther side of it, the mouth of the sac lies over the stone, and, on downward pressure being made, will open to receive it. This method, however, is far inferior in precision and facility of execution to the following method, which I would recommend in preference:—

Place the stone and the fingers in the positions recommended for the process first described; that is, the stone lying immediately behind the opening in the bladder, with its long diameter at right angles to the direction of the opening; the index finger of the right hand introduced through the wound, and resting on the stone, and the fore and middle fingers of the left hand in the rectum, ready to press upon the stone from behind. Let the finger of the right hand be now withdrawn, and the instrument introduced in its place. The stone is now pressed by the fingers of the left hand against the two wires forming the mouth of the sac, and there separating, the stone is forced into the sac itself and secured by the shutting of its mouth. The two slender wires of the sac add little to the bulk of the stone, so that any difficulty experienced in extracting it can depend only on a disproportion between the size of the wound and the stone which is to pass through it; and if that cannot be overcome by address and moderate traction, it must be met by the enlargement of the wound.—*Glasgow Medical Journal*, July, 1858.

